# *Clostridium perfringens* as Foodborne Pathogen in Broiler Production: Pathophysiology and Potential Strategies for Controlling Necrotic Enteritis

**DOI:** 10.3390/ani10091718

**Published:** 2020-09-22

**Authors:** Zuamí Villagrán-de la Mora, María Esther Macías-Rodríguez, Jenny Arratia-Quijada, Yesica Sughey Gonzalez-Torres, Karla Nuño, Angélica Villarruel-López

**Affiliations:** 1 Departamento de Ciencias de la Salud, Centro Universitario de Los Altos, Universidad de Guadalajara, Av. Rafael Casillas Aceves 1200, Tepatitlán de Morelos 47620, Mexico; blanca.villagran@academicos.udg.mx (Z.V.-d.l.M.); sgonzalez@cualtos.udg.mx (Y.S.G.-T.); 2 Departamento de Farmacobiología, Centro Universitario de Ciencias Exactas e Ingenierías, Universidad de Guadalajara, Blvd. Gral. Marcelino García Barragán 1421, Olímpica 44430, Guadalajara, Mexico; mesther.macias@academicos.udg.mx; 3 Departamento de Ciencias Biomédicas, Centro Universitario de Tonalá, Universidad de Guadalajara, Nuevo Perif. Ote. 555, Ejido San José, Tateposco 45425, Tonalá, Mexico; jenny.arratia@academicos.udg.mx

**Keywords:** *C. perfringens*, necrotic enteritis, pathophysiology, control strategies

## Abstract

**Simple Summary:**

*Clostridium perfringens* (Cp.) is an important microorganism from a clinical, food and veterinary point of view. In humans, it is the causal agent of foodborne diseases, commonly associated with the consumption of chicken meat, while, in broilers, it causes clinical or subclinical necrotic enteritis. Cp. has the ability to synthesize toxins, bacteriocins, and enzymes of different nature, which modify the anatomical structure of the intestinal mucosa, enterocytes, and the cellular matrix altering the physiological activities of the gastrointestinal tract, resulting in gastrointestinal disorders, diarrhea, and if it is not attended, death, resulting in significant economic losses for the poultry industry. Food additives such as probiotics, prebiotics, synbiotics, essential oils, organic acids, and enzymes have been presented as alternatives to mitigate the incidence of necrotic enteritis (NE) in broilers, by improving the overall intestinal health and producing healthy birds for consumption. It is imperative to conduct further research on alternatives and efficient products to modulate the intestinal microbiota, and to know the role they play in the immune system, complementing the current demand, economic gain, and keeping the ecology.

**Abstract:**

*Clostridium perfringens* (Cp.) is the cause of human foodborne desease. Meat and poultry products are identified as the main source of infection for humans. Cp. can be found in poultry litter, feces, soil, dust, and healthy birds’ intestinal contents. Cp. strains are known to secrete over 20 identified toxins and enzymes that could potentially be the principal virulence factors, capable of degrading mucin, affecting enterocytes, and the small intestine epithelium, involved in necrotic enteritis (NE) pathophysiology, also leading to immunological responses, microbiota modification and anatomical changes. Different environmental and dietary factors can determine the colonization of this microorganism. It has been observed that the incidence of Cp-associated to NE in broilers has increased in countries that have stopped using antibiotic growth promoters. Since the banning of such antibiotic growth promoters, several strategies for Cp. control have been proposed, including dietary modifications, probiotics, prebiotics, synbiotics, phytogenics, organic acids, and vaccines. However, there are aspects of the pathology that still need to be clarified to establish better actions to control and prevention. This paper reviews the current knowledge about Cp. as foodborne pathogen, the pathophysiology of NE, and recent findings on potential strategies for its control.

## 1. Introduction

*Clostridium perfringens* is a Gram-positive, anaerobic, nonmotile rod that forms subterminal spores. The size of the bacillus on the environment where is found, for example, in culture media for sporulation based on starch the bacillus is long. Meanwhile, in media rich in glucose the bacillus is short. Vegetative cells are relatively cold resistant, and their spores are heat resistant [1,2]. *C. perfringens* can hydrolyze gelatin and reducing nitrates to nitrites; in sulphite media, it generates black colonies due to sulphite reduction. A characteristic test is the lactose fermentation produced by this microorganism, known as *stormy lactose fermentation in milk* due to the large amount of gas it generates [3,4]. This bacterium can develop under microaerophilic conditions due to its ability to produce high amounts of the enzyme superoxide dismutase [5,6]. Its ability to form spores allows it to be ubiquitous and can be found in the environment [7,8].

Beef and poultry, as well as other meat products, are the most important vehicles for this microorganism [9,10,11], although it has also been recovered from vegetables [12] and spices [13]. Butler et al. (2015) [14] described the transmission of *C. perfringens* through water by contact with animals and transmission from person to person. Considered a natural inhabitant of the gastrointestinal tract, the main source of contamination towards meat is fecal matter [15].

According to data reported by the CDC (2019) [16], *C. perfringes* is one of the five pathogens that most frequently cause foodborne illnesses in the United States, ranking second among the etiological agents identified, and, in Australia, it is considered one of the bacteria causing outbreaks [17].

The consumption of chicken meat is important worldwide and a 13% increase in its production is estimated for the year 2027 (OECD-FAO, 2017). In animal production, approximately 70% of the total cost is attributable to the feed. The diets for farm animals contain antibiotics or growth promoters that seek to improve the productive parameters on the farm; however, there is a tendency to use them less frequently, seeking to replace them with what is currently known as sustainable animal diets [18]. 

It is important to mention that some pathogens that cause disease in chickens can be transmitted to humans through their consumption. *Salmonella, Campylobacter jejuni*, and *C. perfringens* are the most studied so far. *C. perfringens* is the cause of subclinical necrotic enteritis in broilers, producing toxins and is the cause of disease in humans [9,19]. 

## 2. *C. perfringens* as a Foodborne Pathogen 

*Clostridium perfringens* can produce a large amount of toxins (Table 1). Toxinotypes of *C. perfringens* cause different diseases in both humans and animals, ranging from subclinical manifestations to serious, life-threatening diseases (Table 2) [20]. 

These diseases are mediated by one or more *C*. *perfringens* toxins [21,22]. Enteric infections in humans and animals have been shown to be associated with *C*. *perfringens* type C [23,24], while the other type of toxins have been confirmed to cause disease in humans or animals, but not both (Table 2). Of the seven *C*. *perfringens* toxin types described, type A is the most frequently identified strain [12,25]. However, type F is the one that causes food-related poisoning in humans [21,26].

The diversity of toxins produced by *C*. *perfringens* has allowed it to be the cause of various diseases in humans and animals. In humans, it is associated with diseases related to food consumption that has been prepared or preserved in inadequate hygienic conditions [17,28]. This type of illness is usually characterized by watery diarrhea and abdominal pain, without fever or vomiting, and the symptoms disappear after 12 to 24 hours [29]. Non-food associated diarrhea due to *C*. *perfringens* has also been described, which usually occurs after a treatment with broad-spectrum antibiotics, and it is common in older adults. It is worth mentioning that this type of diarrhea usually last longer than those associated with contaminated food [30]. Another symptom is necrotic enteritis (NE) caused by *C*. *perfringens* type C [31]. Myonecrosis due to *C*. *perfringens* (also known as gas gangrene) is another condition that can occur in people because of wound infection, generating significant pain, gas accumulation at the site of infection and extensive muscle necrosis, which can put people’s life at risk [32,33].

The toxin of this bacterium also affects some animal species. For example, in broilers the toxin causes necrotic enteritis, which could lead economic losses. The role of the necrotic enteritis B-like toxin (NetB) present in G strains causes NE, which is more frequent in chickens fed wheat or barley-based diets than in those fed with corn [34,35], due to the difference in clostridia proliferation in the diets resulting in a higher number of bacteria in the intestine, as well as a lack of fluidity and digestion, generating an increase in the incidence of NE in chickens and increasing the viscosity of the intestinal contents, mucus production, and growing bacteria [35,36].

## 3. Necrotic Enteritis Pathophysiology

*Clostridium perfringens* is a bacterium found in the gastrointestinal tract of broilers and is acquired from environmental sources such as water, food, or any part of the farm producing these birds, being part of their microbiota [37]. However, a high enumeration number of this microorganism and the presence of toxins in some strains can cause different types of pathologies, among them necrotic enteritis (NE). It is important to mention that an elevated enumeration of *C*. *perfringens* by itself is not the cause of NE but must be accompanied by one or more predisposing factors to develop clinical signs and lesions of the pathology. Enumerations of 0 to 10^5^ CFU/g of *C*. *perfringens* have been observed in the intestine of healthy chickens, while animals with NE report enumerations of 10^6^–10^8^ CFU/g, besides the presence of bacteriocins, adhesins, proteolytic enzymes, collagenolytic enzymes, necrotic toxin enteritis B-like (NetB) and tpeL [38,39].

Currently, the NetB toxin is considered the determining factor inducing NE in birds [40,41]. This 33-kDa toxin is a member of the family of pore-forming toxins with a beta barrel structure encoded by the *netB* gene located in an 85 kb plasmid. The toxin production is stimulated when the *C*. *perfringens* concentration is higher than 10^9^ CFU/g and the bird has low food bioavailability, has a high consumption of polysaccharides, dysbiosis or has suffered intestinal damage. This damage could be caused by coccidial pathogens of the *Eimeria* species, and their colonization causes the release of plasma proteins to the gastrointestinal tract lumen, including more than 11 amino acids, growth factors, and vitamins; they will supply the growth substrate for *C*. *perfringens* [42,43,44].

When *C*. *perfringens* enters the gastrointestinal tract of the bird and encounters a favorable environment, it secretes adhesins and proteolytic enzymes that exert their action on the intestinal mucosa and the surface of the intestinal epithelial membranes, due to their composition. The intestinal mucosa contains mucin binding sites for bacterial adhesins and O-glycosylated glycoproteins that will be degraded by chitinases to provide energy substrates for bacteria. At the same time, *C*. *perfringens* can secrete the bacteriocin perforin, which will inhibit other strains of *Clostridium*, allowing it to have greater bioavailability of nutrients and damage the intestinal mucosa [43,44,45,46,47,48].

Besides colonization and degradation of the intestinal mucosa, the NetB toxin will generate pores to access the enterocytes, and at the same time, adhesins and enzymes capable of degrading collagen of the cell matrix are secreted, which together will allow for the colonization and will determine the NE appearance. NetB toxin production is positively regulated by the VirR/VirS two-component phosphorelay system and by the Agr-type quorum sensing system, the latter being responsible for mediating the regulation of genes involved in phospholipid metabolism and adherence [49,50]. In addition, the phosphorelay system regulates the production of sialidases or neuraminidases with the capacity to hydrolyze the α-glucosidic bond of terminal sialic acid in host glycoproteins and glycolipids, to produce free sialic acid that can be used as a carbon source [45,51], nitrogen, amino acids and energy, as it is metabolized to fructose 6-P by the pathogenic microorganism. They also participate in bacterial adhesion by modifying the epithelial surface and exposing receptors on the enterocyte membrane. Subsequently, *C*. *perfringens* adheres to extracellular matrix compounds such as type III, IV and V collagen, fibrinogen and vitronectin, to later secrete collagenolytic enzymes and hydrolyze them. Adhesion to the extracellular matrix occurs through the fimbrial adhesins of NetB-positive strains [43,44,46,48]. The primary changes occur in the basolateral membrane of the enterocytes, to finally produce necrosis at the level of the mucosa as a result of the destruction of the lamina propria, interruption of intercellular junctions and changes in the extracellular matrix, thus leading to cellular death [45,46].

The *netB* gene along with 36 additional genes, including those that code for two glycohydrolases, two leukocidins, chitinases, an internalin-like protein, a metalloprotease, and several adhesin-like proteins, is located in a plasmid of approximately 85 kb that encodes the pathogenicity loci (NELoc-1, 42 kb), which has been specifically harbored by bird isolates with NE. The high conservation degree of the sequence of this and other identified plasmids (NELoc-2 and NELoc-3) suggests that these come from a recent evolutionary event through conjugative transfer. In accordance with these findings, it is assumed that various virulence factors participate in NE development, whose genes are grouped in pathogenicity loci, some of which are harbored in plasmids [52].

**Figure 1:**
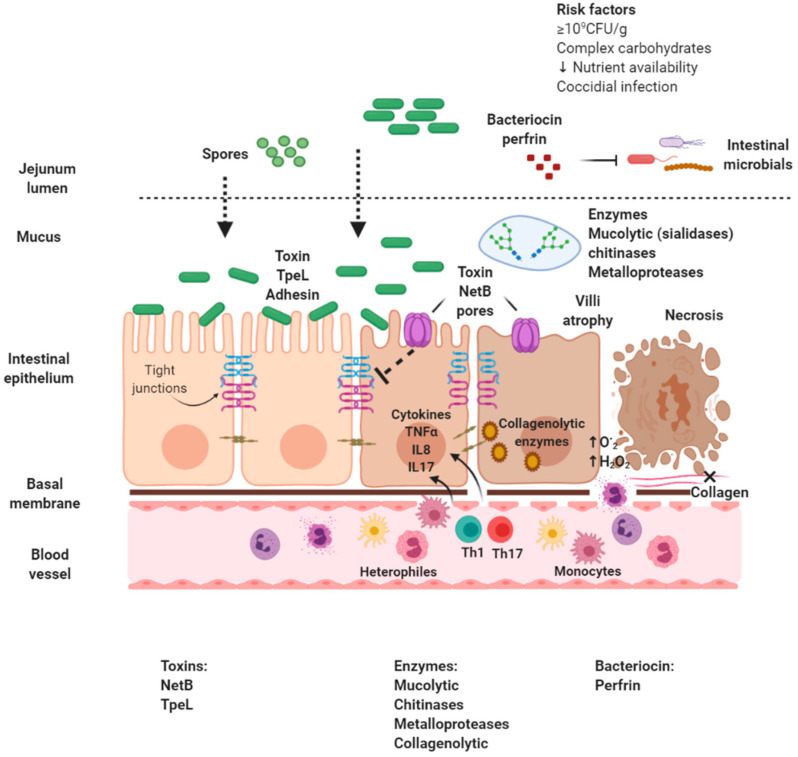
Pathophysiology of necrotic enteritis caused by necrotic enteritis B-like toxin (NetB)-positive *Clostridium perfringens*. Created with BioRender.com

The structural analysis of NetB shows that the interaction domain and binding of the protein with membrane lipids is rich in aromatic amino acids, being essential amino acids R230 and W287, and structurally differs from other proteins of the hemolysin family, substantial for oligomerization of residue S254, suggesting that NetB has a different binding mechanism to membrane receptors [45]; according to some experiments, it is suggested that it binds to membrane cholesterol [53]. Once NetB is secreted in a soluble monomeric form, it binds to the cell surface through the RIM domain and subsequently oligomerizes, producing a pre-heptameric pore in the lipid bilayer. This oligomerization process induces conformational changes in the protein to generate a barrel structure with antiparallel β-sheets and forms a mushroom-like transmembrane pore with a subsequent alteration of membrane permeability [53,54]. The heptameric pore formed at the plasma membrane level by the NetB toxin has an internal diameter of 26 Å, with a hydrophilic nature, which favors the destabilization of the ion flow by allowing the exit of K^+^ ions and the entry of Ca^2+^, Na^+^ and Cl^-^ (showing preference for cations), producing osmotic cell lysis [22,41,55]. As intracellular calcium increases, the cascade of events for necrosis programming is influenced by the activation of calpain and cathepsin secretion from lysosomes. In addition, an alteration in the mitochondrial activity is observed with an increase in reactive oxygen species and a decrease in ATP [56]. Free radicals can accumulate in the mitochondria and uncouple the proteins of the mitochondrial inner membrane, leading to a decrease in ATP levels, with losing the integrity of the intercellular junctions in the gastrointestinal epithelium, increasing the permeability of the mucosa and, finally, cell death (Figure 1) [56,57].

In the conserved region of the plasmid NELoc-1 that codes for the NetB toxin, there is also the gene that codes for the zinc metalloprotease, ZmpA, which, together with another metalloprotease, ZmpB, has been implicated in NE in chickens. Such proteins have high binding affinity for the mucin glycoprotein, a constituent of the mucosa of the gastrointestinal epithelium. Such metalloproteases participate in the development of NE, since the *zmpA* gene has been identified in isolated strains of birds with the disease, although the *zmpB* gene was still identified in isolates of birds in the absence of the disease, the lack of one or both genes generate strains with reduced virulence, which is why they are presumed to participate together in pathogenesis’s development [47].

Likewise, it has been observed that the expression of the *tpeL* gene occurs during sporulation and the TpeL toxin is secreted to promote the adhesion of *C*. *perfringens* type A in epithelial cell cultures [58]; disease and mortality are induced more rapidly in birds infected with TpeL-producing strains, which could potentiate the effect of other toxins like NetB [59]. The binding and entry of this toxin into the enterocyte is mediated by the endocytic receptor Lpr1 [60].

### 3.1. Clinical Alterations

Chicken’s intestinal health is determined by the balance between anatomical components and their physiological activities. However, the development and maturation of the gastrointestinal tract is generated through the bird’s life, as well as the exposure to different environmental variables, which will establish the morphological changes and the specific activities of each intestinal segment. Thus, the mucosa and intestinal villi with their microvilli are necessary for the adequate absorption of nutrients and the establishment of the intestinal microbiota, while the gut-associated lymphoid tissue (GALT), together with the mucosa and microbiota, provides an immune complex that will work as a gastric defense mechanism [61,62,63]. Therefore, an aggression to any of these components, especially in the first weeks of life, could trigger an alteration in the integrity of the intestinal epithelium, in the bioavailability and absorption of nutrients, promoting a bacterial dysbiosis and an intestinal inflammatory process.

Due to the above, the chicks, being immunologically and physiologically immature, are more susceptible to be infected by *C*. *perfringens* (NetB positive), either clinically or subclinically. The clinical phase of disease caused by *C*. *perfringens* infection is called NE and is characterized by a sudden increase in mortality of up to 50% of the population [44,64]. The main characteristic is necrosis at the intestinal level, whose clinical signs include depression, dehydration, drowsiness, diarrhea, and a decreased food consumption. Likewise, lesions are observed throughout the gastrointestinal tract mainly in the jejunum, ileum, expanding to the cecum and duodenum, with a thin, dilated wall and with the presence of gas. The mucosa is gray-brown or yellow-green, and, occasionally, lesions occur in other organs like the cecum, liver, and kidney [42,56,64].

At the microscopic level, in the early stages of the disease, a hyperemic lamina propria is observed, with infiltration of heterophiles, lymphocytes, and plasmatic cells, edematous areas and structural alterations. Likewise, villi flattening, and the congestion of blood vessels are observed in the lamina propria and submucosa. Subsequently, there is necrosis in the mucosa and villi, a pseudomembrane with a tissue fragment, fibrin presence with cellular adhesions to the gastric mucosa, where there are bacterial conglomerates. In the later stages of the disease, blood vessels, liver and kidney are affected, accompanied by red cell changes and necrosis in follicular lymphocytes [42,44,56,64].

While subclinical pathology has several non-specific signs, such as poor digestion, low weight gain, increased feed conversion ratio, and an increased risk of mortality, lesser-grade histopathological lesions can be observed in the intestinal tract, including ulcers, bile duct hyperplasia, and inflammation. The chronic subclinical disease process allows bacteria to reach the bile duct and bloodstream, therefore, the pathogen can be found in the liver [42,65,66]. Because the subclinical process does not have exacerbated clinical manifestations and high mortality, many birds do not receive treatment, which leads to severe economic losses.

### 3.2. Immune System Activity

Part of the clinical picture of the disease is due to the action of the immune system against the aggression exerted by the virulence factors of *C*. *perfringens*. The first step is observed in the intestinal mucosa, whose degradation allows access to nutrients and pathogen colonization; therefore, bacterial accumulations are observed in this segment and a decrease in the thickness of the intestinal mucosa. 

Subsequently, the formation of the transmembrane pore in the enterocyte and the alteration in the extracellular matrix by the collagenolytic enzymes of *C*. *perfringens* will affect the tight junctions and their components, as well as the binding proteins claudins, occludins, molecules of junctional adhesion molecules (JAMs), coxsackie virus and adenovirus (CAR) receptors, and tricellulins, until the damage compromises the integrity of the lamina propria [42,45,67]. This is accompanied by the activation of the mucosal immune response to increase epithelial permeability. Thus, pro-inflammatory cytokines, such as TNF, IL-1-, and LIGHT (tumor necrosis factor superfamily member 14), promote the dysfunction of the barrier generated by tight junctions by inhibiting the transcription of binding proteins and inducing the redistribution of occludins, ZO-1 and claudins-1 through the dynamics of the cytoskeleton. Additionally, cytokines promote the transcription of MLCK kinase (Myosin Light-chain kinase), which activates myosin II by phosphorylation, which leads to the reorganization of tight junction proteins and even promotes endocytosis of the binding complex from the apical zone of the enterocyte, thus altering paracellular permeability [57]. The loss of the tight junction integrity results in a leaky gut, altering the passage of solutes in the transmembrane, affecting the cytoskeleton and function of the enterocyte, or giving way to microorganisms or their components, such as lipopolysaccharides to circulation (endotoxemia). This compromises the epithelial function, the structure of the apical and basolateral barrier of the enterocyte, causing diarrhea besides activating the gastric immune system, resulting in local and, later, systemic inflammation [67,68].

During the inflammatory process, there is activation of CD4, Th1 and Th17 lymphocytes, whose inflammatory cytokines promote the recruitment of heterophiles, monocytes, and lymphocytes, as well as their translocation and migration to the damaged site. Therefore, its accumulation results in an inflammatory process accompanied by the flattening of villi and hyperplasia of the crypts, which, together with the degradation of the gastric mucosa and alteration of the enterocyte, leads to a decrease in the absorption surface and diarrhea [56,67,69]. Villi flattening decreases the site of absorption of macro and micronutrients that impact on the health status of the bird, which can present mal absorption, malnutrition, food deficiencies and pathologies related to nutrition such as anemia, low weight and low feeding efficiency [62,70].

The effect on the enterocyte and tight junctions, caused by the NetB toxin and collagenolytic enzymes, is enhanced by the presence of other inflammation mediators, since these exert their action on vascular permeability, which causes overexpression of oxygen reactive species and capillary congestion, leading to edema and the possible presence of hemorrhages, and necrosis [42,63,71]. The increase in vascular permeability at the injury site caused by inflammatory mediators such as histamine, leukotrienes, prostaglandins, among others, stimulating endothelial cells to express adhesion molecules in the basement membrane, which allow the anchoring of heterophiles and platelets. These cells are exposed to inflammatory mediators and are activated to release oxidant molecules and proteases (elastase), in addition to cytokines like TNF and IL-1β, which damage the endothelium and microvasculature by increasing the inflammatory response recruiting more leukocyte cells [72]. 

Heterophiles transmigration between endothelial cells disrupts inter-endothelial junctions, and in conjunction with the reorganization of tight junction proteins promoted by TNF and IL-1β, there is a considerable increase in vascular leakage [57,72]. Macrophages located in the lamina propria, the submucosa, and the intestinal lymphoid organs are among the first cells of the epithelium to respond to infection. Activated macrophages produce cytokines TNF, IL-8 and IL-1β; in addition, they can produce nitric oxide, which has a vasodilator and antimicrobial effect. IL-8 promotes the attraction of lymphocytes, the activation and degranulation of heterophils, in response to mediators such as cyclooxygenase-2 and 5-lipoxygenase that produce potent vasoactive and pro-inflammatory effects by activating endothelial cells, neutrophils, and platelets [72].

The extracellular matrix of an inflamed tissue is composed of fibronectin, fibrinogen, and vitronectin, which are deposited in the tissues as a result of plasma extravasation and by protein synthesis, from stromal cells, in response to the activation of the inflammatory mediators and adhesion of the heterophiles, which, when degranulated, release proteases with fibrinolytic activity, with the consequent deposition of fibrin in the injured tissue. For their part, activated mast cells release histamine, 5-hydroxytryptamine, proteases, heparin, cytokines and other inflammatory mediators from their granules, which increase vascular permeability, generate vasodilation, alter intestinal motility, promote epithelial cell secretion, with the consequent increase in transit, and the expulsion of intestinal content [72].

The loss of mucosa, the flattening of villi and the alteration of intestinal permeability generate a change in the site of action and the available nutrients of the intestinal microbiota, and with it an alteration in the bacterial communities and their metabolic and immunological effect [73]. Dysbiosis is mainly seen in bacterial groups such as *Ruminococcus*, *Clostridium*, and *Lactobacillus*. The first is found in a higher percentage in the cecum with a metabolic activity that includes the production of butyric acid. Gharib-Naseri et al. [38] reported that low *Ruminococcus* enumerations have been observed in chickens with NE, which could cause a decrease in the main energy metabolite of the intestinal epithelium, butyrate, which decreases blood flow that is linked to nutrients absorption, reducing cell proliferation, mucin production, as well as the defense mechanisms and anti-inflammatory activity of IL10 [63].

On the other hand, it has been observed that in chickens that have been challenged, *Lactobacillus* enumerations, particularly in cecum, are higher compared to controls. This could be due to disturbances in the bioavailability of nutrients in the models, the increase due to the recovery of the chickens after NE or due to the over-influx of the ileum to the cecum due to the microbial challenge and that is related to a greater amount of acid lactic in blind. On the other hand, it has been reported that in animal models that were infected with *C*. *perfringens*, said pathogen displaces or inhibits the native microbiota, particularly the Clostridial community, whose proportion in healthy birds is represented by *Clostridium proponicum*, *Clostridium leptum*, and *Ruminococcusbromii*. Competition between clostridials probably allows bacteria such as lactobacilli to increase their enumerations and the overpopulation of other less dominant species [38,74,75]. 

## 4. Detection Mechanisms

### 4.1. Histopathological Detection

Evaluating the damage of the disease has been carried out through biological models. To do this, the presence of colonies of Gram-positive bacilli is observed, and a score has been proposed that allows a semi-quantitative evaluation, whose criteria include the observation of macroscopic and microscopic damage to the epithelium, mucosa, and reliability of the intestine and gas accumulation [70].

After histopathological evaluation, it is common to find necrotic enteritis (NE) lesions in the proximal region of the jejunum (between the distal end of the duodenum and Meckel’s diverticulum), anywhere in the small intestine, as well as in the cecum and/or the colorectal region (Table 3).

Occasionally, multifocal coagulative necrosis lesions can be found in the liver and bile ducts, with the presence of exudative fibrin and Gram-positive bacilli; the tissue appears thickened and with granulomatous inflammation [64]. The gross lesions that occur in the NE are recorded according to a scale of tissue damage (Table 4).

Birds that die from NE undergo a rapid decomposition, the intestine begins an autolysis process, which makes the histopathological analysis difficult; thus, the diagnosis requires further evaluation.

### 4.2. Immunological Detection

Through an ELISA-type immunosorbent assay, it has been possible to detect the presence of high levels of CPA toxin in intestinal samples from chickens with NE and/or serum anti-CPA [64] or anti-NetB [79] antibodies, the latter reflecting a clinical or subclinical picture of the disease. It is worth mentioning that toxins can be degraded by proteases, or be produced after death, so their detection is not conclusive for NE.

Recently, a method has been developed to detect the levels of IL-10 in serum of infected chickens and intestinal epithelial cells stimulated with *C*. *perfringens*, with an ELISA to capture antigens by mouse monoclonal antibodies against chicken IL-10, representing a useful tool to monitor the disease [80].

### 4.3. Molecular Detection

*C*. *perfringens* detection in samples from the gastrointestinal tract of chickens can be performed by quantitative real-time PCR using a fluorogenic assay, with a hydrolysis probe (5′ nuclease) for the detection and quantification of specific 16S rDNA sequences for *C*. *perfringens* obtained from the gastrointestinal contents of chickens [81]. The pathogenic strains can be detected through the identification of genes coding for the relevant toxins in the *netB* and *tpeL* pathogenesis of NE in isolated clinical samples, with a specific multiplex PCR, thus allowing for more efficient sampling and diagnosis [43,82].

Although the detection of pathogenic strains of *C*. *perfringens* may be simple, the diagnosis of NE as such is not possible in a timely manner, since it is feasible until the bird has died. Among the strategies used for raising healthy animals is the use of various methods of disease control through the incorporation of compounds in the diet that contribute to modulating their nutrition. The diet significantly affects the intestinal microbiota of broilers and is responsible for regulating important aspects such as immune and metabolic response.

## 5. Control 

The intestinal microbiota of broilers constitutes a crucial factor in modulating the immune response and productive efficiency. However, its composition is affected by the diet supplied and the incorporation of food additives (antibiotics or other growth promoters) to improve the productive parameters on the farm, causing alterations that favor the development of pathogens such as *C. perfringens*. Currently, there is a trend to replace the use of antibiotics with what is known as sustainable animal diets [18]. 

The basic strategies used to control necrotic enteritis (NE) in broilers are the reduction of pathogens and modification of diets and/or feed additives [82]. The first strategy usually involves establishing biosecurity and sanitation protocols on farms. For its part, the nutritional approach includes the use of probiotics, prebiotics, symbiotics, phytogens, organic acids, and dietary modifications, which are discussed below.

### 5.1. Probiotics

The incorporation of probiotics in diets has been considered as a promising alternative to the use of antibiotics and growth promoters. Probiotics have been defined as "live microorganisms which, when administered in adequate amounts, can confer benefits to the health of the host" [83].

The benefits attributed to the probiotics incorporated in broilers diets are diverse and include: (1) modulation in the composition of the intestinal microbiota through the production of pathogen growth inhibitory metabolites; (2) improving food efficiency conversion and, therefore, a significant increase in production performance, in addition to showing improvement in meat quality; (3) stimulation of the immune system, increasing the levels of immunoglobulins in serum, specifically IgG (or IgY) and IgA and the secretion of IgA in mucous membranes (sIgA), while reducing the severity of pro-inflammatory processes, and (4) contribution to the improvement in the safety of raw meats destined for human consumption by competitive exclusion mechanisms and/or by neutralization of toxins [84,85,86,87,88,89].

Probiotics are widely used microorganisms to deal with specific diseases such as avian subclinical NE. The efficacy of probiotics belonging to the genera *Bacillus*, *Lactobacillus*, *Enterococcus*, *Bifidobacteria*, and *Saccharomyces* has been evaluated both in vivo and in vitro [90]. However, in in vivo tests using strains of the *Bacillus*, *Lactobacillus*, and *Enterococcus* genera, their beneficial effects have been described in greater depth (Table 5). One of these studies performed a meta-analysis that included independent trials carried out in different countries simultaneously, demonstrating in large-scale evaluations that the supplementation of probiotics like *B. subtilis* DSM32315 significantly improves productive parameters and decreases the histological damage caused by *C. perfringens* [91].

The composition of the microbiome associated with broilers has been correlated with improved production efficiency, alluding to the fact that the use of probiotics represents a viable alternative to avoid the use of antibiotics in diets [92]. It has been suggested that probiotics may beneficially affect the structure of the host gut microbiota, consequently improving the growth and survival of farm organisms [86]. The main effects described in animals whose diets were supplemented with probiotics are related to an increase in the enumerations of *Lactobacillus*, *Bifidobacterium*, and *Butyricicoccus* and a decrease in *Escherichia coli*, *C. perfringens*, and *Staphylococci*. Stanley et al. (2016) [27] identified a significant correlation between the presence of *Faecalibacterium prausnitzii*, feed conversion and metabolizable energy in broilers ceca microbiota, while the genus *Lactobacillus* was correlated with a high level of feed intake and a low feed conversion [93]. In other studies, the efficacy of *L. johnsonii* FI9785, a producer of a heterologous endolysin, was observed in vitro and in vivo reducing *C. perfringens* as a way to improve the safety of chicken meat for human consumption [94,95] (Table 6). 

In addition to the above, the concept of competitive exclusion has been described, which was born from the work carried out by Rantala and Nurmi in 1973, who proposed the inclusion of bacteria isolated from adult chickens to prevent *Salmonella Infantis* colonization [113]. This term raises the possibility of “implanting a healthy microbiota” in the first days of the animal’s life, and thus preventing colonization by pathogens [114]. For this, commercial products (Aviguard^®^, BROILACT^®^, PoultryStar^®^, MSC™) have been suggested with an effect against *C. perfringens*, which causes NE in broilers [114]. However, only MSC™ was evaluated to decrease the enterotoxin produced by *C. perfringens*, suggesting its usefulness against the incidence of NE in chickens and reducing the risk of disease in humans [115].

Due to the above, the possibility of an early programming to modulate the intestinal microbiota has been considered as a potentially useful strategy to improve health, well-being and productivity in broilers through probiotics, by reducing pathogen enumerations in the gastrointestinal tract of chickens and, with it, the risk of contamination to contribute to the safety of raw meats [116,117]. 

### 5.2. Prebiotics

The International Scientific Association for Probiotics and Prebiotics defines prebiotics as “a substrate that is selectively used by host microorganisms, conferring health benefits”; when administered orally, these are called dietary prebiotics [118]. 

A good prebiotic should meet the following characteristics: 1) resist exposure to gastric acid, it should not be hydrolyzed or absorbed in the upper part of the gastrointestinal tract; 2) serve as a selective source of nutrients that support growth and/or metabolic activity of beneficial host members of the gut microbiota, and 3) induce luminal responses or other systemic physiological responses that benefit the host in some way [119]. Thus, the compounds that meet these characteristics are indigestible oligosaccharides or polysaccharides [120], also named refined functional carbohydrates [118], such as mannan-oligosaccharides (derived from the cell walls of *Saccharomyces cerevisiae*), β-glucans (derived from cell walls of fungi or yeasts), galacto-oligosaccharides and fructo-oligosaccharides like inulin, levan and branched groups (extracted from different plants, hydrolyzed from polysaccharides or produced by microorganisms) [121], being inulin and fructooligosaccharides the most used in the poultry industry [122], with a degree of polymerization of two to twenty monomers [123].

The use of prebiotics in poultry production systems is based on the fact that they are able to improve the intestinal epithelium (longer villi and shallower crypts) [124] and feed conversion and efficiency [125] through the synthesis of metabolites from their fermentation, such as short-chain fatty acids [126], mainly acetate, propionate, and butyrate, which are absorbed directly from the hindgut and used as an energy source in tissues [127], which in turn promote weight gain and performance [125].

Moreover, they improve the mineral absorption, specially Ca and P, when administered at a rate of 10 g/kg of feed, which in turn impacts bone mineralization in broilers [120], promoting a symbiosis in the intestinal microbiota, increase intestinal colonization of lactic acid bacteria and are capable of inhibiting intestinal colonization of pathogens, thereby restricting the amount of toxic metabolites generated by them (ammonia, indoles, phenols, and thiols) [128]. They also reduce the intensity and time of histopathological conditions caused by *C. perfringens* in the jejunum and duodenum [124,126].

The effects obtained are dependent on the quantity, type and origin of the administered prebiotic, as well as on the characteristics of the birds (breed, sex, age) and the environment (hygiene, house maintenance, environmental stress, temperature) [120,129].

### 5.3. Synbiotics

The term synbiotic was used for the first time in 1995 by Gibson and Roberfroid when referring to “a mixture of probiotics and prebiotics that can beneficially affect the host by improving the survival and implantation in the gastrointestinal tract of live microorganisms supplemented in the diet, by a selective stimulation of the growth and/or activation of the metabolism of one or a limited number of health-promoting bacteria, and therefore, improving the well-being of the host” [130].

The main reason for using a symbiotic is that the probiotic without the prebiotic will have less chance of surviving in the gastrointestinal tract, as it will show less tolerance to temperature, oxygen, and low pH. In addition to the above, the administration of a synbiotic improves the survival of the probiotic during its passage through the upper gastrointestinal tract [122,131]. Among the benefits of using synbiotics are: (1) raising the levels of lactobacilli and bifidobacteria, as well as the balance of the intestinal microbiota; (2) improving immunomodulation; and (3) preventing bacterial translocation [132]. In broilers, dietary supplementation with synbiotic products has been reported to significantly improve body weight, average daily weight gain, feed efficiency and percentage of body mass yield compared to the controls or chickens fed only with probiotics [133].

There are some commercial synbiotic products intended for the chicken meat industry; among them are: Biomin^®^IMBO (ME BIOMIN GmbH) made up of *Enterococcus faecium* and fructooligosaccharides (FOS), and PoultryStar^®^ (ME BIOMIN GmbH), which includes a mixture of *Bifidobacterium animalis, Enterococcus faecium, Lactobacillus reuteri, L. salivarius, Pediococcus acidilactici* and inulin, and Synbiotic poultry (Vetafarm) containing *L. acidophilus, L. casei, L. salivarius, L. plantarum, L. rhamnosus, L. brevis, Bifidobacterium bifidum, B. lactis, Streptococcus thermophilus* and inulin [134].

Synbiotics have been evaluated in the poultry industry to eliminate or decrease intestinal counts of specific pathogens such as *Campylobacter jejunio*. Supplementation of a mixture of *Bifidobacterium longum* subsp. *longum* PCB133 and xylooligosaccharides demonstrated their efficacy in reducing the pathogen through the alteration of the intestinal microbiota when it is developing [135].

Few studies have addressed the use of synbiotics as a strategy to decrease the severity of necrotic enteritis (NE) caused by *C. perfringens*. Among the most important results, a consistent impact has been observed in the reduction of the pathogen enumerations and the severity of the histopathological damage at the intestinal level, in the intestinal damage score and in mortality percentages (Table 7). On the other hand, these studies describe an increase in weight gain, in the enumeration of lactic acid bacteria at the intestinal level, and in the number of specific antibodies at the mucosa level in broilers (Table 7) [136,137].

The results of studies conducted with synbiotics in chickens remain controversial. Some researchers have highlighted the efficacy that they have on the significant reduction of pathogens such as *Escherichia coli* in the cecum content when used combined, strains from the group of lactic acid bacteria and yeasts [136]. For their part, Mookiah et al. [137] did not observe a synergistic effect when combining probiotics with prebiotics (11 *Lactobacillus* strains and isomalto oligosaccharides) in determining microbial populations in cecum or volatile and non-volatile fatty acid concentrations in broilers.

Studying the effect that different synbiotics have on pathogens of sanitary importance such as *C. perfringens* and/or its toxins, and their impact on the safety of meat for human consumption, is a subject that still needs to be explored in more detail.

### 5.4. Phytogenics

Phytogenic additives are components and biologically active substances extracted from plants, such as oleoresins, tannins, saponins, flavonoids and alkaloids, with a positive effect on growth and animal health [141].

Phytogenics increase antimicrobial activity, have antiviral, antioxidant, and anti-inflammatory properties, stimulating the endocrine and immune system. They promote a higher metabolic and immune status in chickens, as well as greater well-being. Several plant-derived compounds have been shown to have beneficial effects on the gut environment and gut microbiota. Its action mechanism is based on altering the permeability of the membrane of microorganisms, causing the leakage of intracellular material. It is difficult to identify its active principle, because there is a variation in growth conditions, climate, harvest, and manufacture, as well as in the biological factors of each plant species [142,143]. Table 8 shows a list of the plants most used for the control of *C. perfringens* in broilers, as well as a description of the effects caused by the phytogenics tested.

### 5.5. Organic Acids 

Organic acids and their corresponding salts or esters are widely used as a feed additive in poultry production. They can vary considerably in their functionality due to the number of carbon atoms and it they are aliphatic or aromatic. They are natural constituents of animal or plant tissues or products of microbial fermentation [143].

Carboxylic acids with an aliphatic chain or fatty acids are classified into short chain fatty acids (SCFAs, 1–5 carbon atoms; C1–C5) and medium chain fatty acids (SCFA, 6–12 carbon atoms; C6–C12). The suggested effects of organic acids are antibacterial activity through pH regulation, changes in the composition of the microbiota, immunomodulatory action, and stimulation of the intestinal mucosa [133,143].

Organic acids have been used as inhibitors of enteric pathogens or as antimicrobials. Their mechanism of action can be by non-dissociation, where, by penetrating the bacterial cell wall, they alter their normal physiology and generate a change in their internal pH, with a dissociation between H^+^ and anions, which leads to energy consumption that puts the growth of the bacteria at risk, even causing death. In addition, they promote changes in the microbiota, have an immunomodulatory action and stimulate the intestinal mucosa. The effects in broilers depend on the base of the organic acid product, dose and type [143,152].

Capric-caprylic, caproic, and lauric acids are associated with improved intestinal histomorphology and decreased stool *C. perfringens* enumerations [149]. On the other hand, hexanoic, benzoic and butyric acids are associated with an improvement in intestinal histomorphology, a decrease in *C. perfringens* counts in the liver and cecum content, and a decrease in the frequency and intensity of intestinal lesions associated with NE [142]. Sodium lauryl lactylate acid has also been reported to prevent and inhibit intestinal colonization by *C. perfringens* [153].

### 5.6. Dietary Modifications and Enzymes

The nutritional content and feed presentation significantly affect the development of NE in broilers. Dietary management is a promising strategy for its control [151]. In this sense, different strategies are considered, among which are dietary restriction, modification of the content and source of macronutrients, [154,155] and the addition of enzymes to the diet [156]. 

Food restriction is applied in poultry, to control the growth rate and prevent metabolic disorders. Its protective effect against NE could be attributed to the stimulation of the immune system, the influence of the endocrine system, a decrease in pH and the viscosity of the intestinal content, promoted by food restriction [157].

With regard to modifications in the content and source of macronutrients, it has been shown that the level and source of dietary protein have a direct effect on the concentration of *C. perfringens* in broilers [155]; this is how those diets high in protein from fish increase the risk of developing NE, for which reason the use of other sources such as soy is currently promoted [158]. Regarding carbohydrates, the administration of whole grains is a frequent practice in poultry rearing, since it is associated with the improvement in productive performance and the general and intestinal health of the birds [159]. The use of whole grains to control NE is based on mechanical stimulation of the gizzard, pH reduction and viscosity of the intestinal content, which together create an unfavorable environment for the proliferation of *C. perfringens* [160].

In broiler production, the use of enzymes derived from microorganisms (fungi and bacteria) through traditional submerged liquid fermentation or solid-state fermentation is common [161]. Enzymes such as proteases, glucanase, manase, cellulase, amylase, phytase and xylanase are added to the feed to overcome the negative effects of non-starch polysaccharides and increase the digestibility, management and absorption of nutrients for poultry [157,162,163]. Furthermore, the proliferation of *C. perfringens* in the gastrointestinal tract of broilers [150,157,161,164], which is achieved by reducing the viscosity of the gastric and intestinal contents (cellulase [164], phytase [161], glucanase [150,157], mannanase [150] and xylase [162,165,166]), promotes intestinal colonization of lactic acid bacteria (glucanase [150,157], mannanase [150] and xylase [162,165,166]) and even improves physical characteristics such as longer villi and fewer deep crypts and functional characteristics such as gut permeability (glucanase [150,157] and xylase [162,165,166]).

As has been reviewed, there are currently different alternatives to the use of growth-promoting antibiotics to control NE; however, these alternatives remain questioned in their efficacy [152]. Although the bacterial microbiota associated with broilers has shown variations in the structure of their communities with regarding control strategies and the influence of pathogenic bacteria, more comparable studies of farm chicken microbiomes are required that consider individual variability and variations between samples of cecal, jejunal or associated mucosal content [167] to deepen and compare the information.

## 6. Conclusions

*Clostridium perfringens* is an important microorganism in the clinical, food and veterinary areas. The diversity of toxins produced by this microorganism not only makes it a risk to human health, but also to animal health. In the latter, the problem is that it causes subclinical diseases that generate great losses, particularly in the poultry industry, because *C. perfringens* is capable of producing various toxins and bacteriocins, some of which have already been identified and characterized. However, other pathogenicity factors cannot be discarded.

Currently, the infection produced in broilers, known as necrotic enteritis (NE), associated with this microorganism, has become a problem to maintaining the health of birds, affecting reproduction and conservation, and the supply for human consumption, due to the fact that the disease occurs subclinically and a diagnosis cannot be made in a timely manner, generating significant economic losses for the producer.

Chicken meat is the most consumed animal protein and enough supply for consumers requires mass production strategies, exacerbating the problem of by infections by pathogens such as *C. perfringens.* Due to this, there is a need to find economical, environmentally friendly and efficient alternatives in the modulation of the intestinal microbiota, which contribute to the efficient production of broiler chicken to meet current and future demand.

The use of various food additives based on probiotics, prebiotics, symbiotics, essential oils, organic acids and enzymes have been presented as various alternatives to mitigate the incidence of NE, achieving an improvement in the general intestinal health of birds, with the opportunity to produce healthy birds for consumption.

Perspectives: It is imperative to carry out more research on alternative and efficient products for the modulation of the intestinal microbiota, in addition to the role they play in the immune system, where consistent positive effects are needed to fulfill the current demand, while keeping a safe environment. It is also important to establish standardized protocols that consider individual and inter-sample variability, and consider the utility of molecular detection mechanisms and epigenetic modifications underlying treatment with alternative products such as essential oils and organic acids where research has not yet been clarified.

## Figures and Tables

**Table 1:** Types of *Clostridium perfringens* according to the toxins produced and the genes that encode the toxins.

	Toxins					
Type	Alpha (α)	Beta (β)	Epsilon (ε)	Iota (ι)	CPE	NetB
	(*plc* o *cpa*) *	(*cpb*) *	(*etx*) *	(*iap* y *ibp*) *	(*cpe*) *	(*netB*) *
A	+	−	−	−	−	−
B	+	+	+	−	−	−
C	+	+	−	−	+/−	−
D	+	−	+	−	+/−	-
E	+	−	−	+	+/−	−
F	+	−	−	−	+	−
G	+	−	−	−	−	+

*Gene for each toxin. Taken from Rood et al., 2018 [21].

**Table 2:** Toxigenic types of *Clostridium perfringens* and their association with diseases in humans and animals.

Type of Toxin	Main Toxin	Diseases that Cause
A	α	Wound infection in humans (gas gangrene or clostridial myonecrosis), necrotic enteritis in birds, ulcerative abomasitis, mild necrotizing enteritis in piglets, and endotoxemia in South American camelids.
	α, CPE	Food poisoning in humans, non-food gastrointestinal diseases in humans, and diarrhea in animals such as dogs, pigs, and foals.
	α, β2	Gastrointestinal disease in swine.
B	α, β, ε	Dysentery and hemorrhagic enteritis in lambs and kids.
C	α, β	Necrotizing enteritis in humans, enteritis in dogs, chickens, and South American camelids.
	α, β, β2	Gastrointestinal disease in swine.
D	α, ε	Enterotoxemia in sheep and goats (pulpy kidney disease).
E	α, ι	Enterotoxemia in rabbits, dogs, cattle, and sheep.
F	α, CPE	Human food poisoning and non-food associated diarrhea.
G	α, NetB	Subclinical necrotic enteritis in chickens.

Bruce et al., 2006; Kiu& Hall, 2018 and Rood et al., 2018 [20,21,27].

**Table 3:** Lesions found on histopathological examination.

Lesion	Characteristics
Necrosis	Mucous discolored, thick, coarse granular texture, moderately firm and adherent or smooth, and moist. Areas of intensely eosinophilic villi covered with clostridia delimited by heterophilic infiltrate with fibrin.
Ulcers	Sunken fossae with rough and reddened exposed surface, crater-like, thinning and detachment of mucosa, shiny appearance. Presence of re-epithelialized ulcers on the serous surface.
Hemorrhage	Limited in the margin of the lesions or in the intestinal lumen.
Thin and flaccid intestinal wall	Detachment of large areas of mucosa that accumulate in the intestinal lumen, loss of smooth muscle tone, presence of discoloration produced by thick dark green bile at the duodenum and proximal jejunum. Smelly gas build-up.

Cooper et al., 2013; Smyth, 2016 [64,66].

**Table 4:** Scale of gross lesions in the small intestine with chronic enteritis.

Number	Lesions
0	No apparent injuries.
1	Thin or brittle wall. Congested intestinal mucosa.
2	Focal necrosis or ulceration (1 to 5 lesions).
3	Coalescent multifocal areas of necrosis (6 to 15 lesion targets).
4	Extent of severe necrosis (more than 16 lesion targets.
5	Necrosis patches 2–3 cm long (variable amount).
6	Extensive diffuse necrosis (variable amount).

Keyburn et al., 2006; Shojadoost et al., 2012; Yang et al., 2019 [76,77,78].

**Table 5:** Probiotics used in the treatment of necrotic enteritis caused by *Clostridium perfringens* in an avian model.

Genus	Strain	Results	Reference
***Bacillus***	*B. amyloliquefaciens* H57	Improvement of feed conversion. Lower score in intestinal lesions caused by *C*. *perfringens*. Structural protection of villi at the mucosal level (improves intestinal integrity).	[96]
	*B. coagulans*	Improvement of intestinal morphology and cecum and liver damage decreases (P ˂ 0.01). Expression increase of fowlicidin-2, an antimicrobial peptide described in chickens. Increased levels of sIgA and alkaline-phosphatase activity in jejunum. Increase in the expression levels of lysozyme in the jejunum. Inhibition of growth, colonization, and invasion by *C*. *perfringens*.	[97]
	*B. licheniformis* H2	Normalization of disorders in the microbiota caused by infection with *C*. *perfringens*.	[98]
		Significant suppression of the negative effects on weight gain, decrease in feed consumption, and feed conversion rate (P ˂ 0.05). Increase in the villis height: depth ratio of the crypts in the ileum (P ˂ 0.05). Increase in the activity of antioxidant enzymes and intestinal capacity in ileum, serum, and liver (P ˂ 0.05). Increase in the concentration of Bcl-2 protein in the liver.	[99]
	*B. subtilis* DSM32315	Meta-analysis carried out in three different countries and five independent trials. Significantly improves weight gain and feed conversion. Decreases mortality. Lower injury score.	[91]
	*B. subtilis* PB6	Lower score of intestinal lesions. Increase in the concentration of propionic acid in cecum.	[100]
	A mix of 6-probiotic strains, 4 *Bacillus subtilis* (CPB 011, CPB 029, HP 1.6, and D 014) and 2 *Bacillus velezensis* (CBP 020 and CPB 035)	Significantly improves feed conversion. Increase in villus height (P ˂ 0.0001) and in the ratio of villi height: crypt depth (P ˂ 0.0004) in duodenum and jejunum.	[101]
***Lactobacillus***	*L. johnsonii* BS15	Significant increase in the levels of IgG (or IgY) and IgA in serum after 21 days. Beneficial effects on subpopulations of T lymphocytes in peripheral blood.	[102]
	*L. plantarum* 1.2567	Significant decrease in the loss of epithelial cells and lymphocyte infiltration, showing an effect of attenuation of the inflammatory response. Significant reduction in intestinal injury scores. Improvement in weight gain. Improvement in the structure of microvilli.	[103]
	*L. fermentum* 1.2029	Attenuation of the inflammatory damage causing distortion in the crypt architecture, infiltration of granulocytes in the lamina propria and subepithelial and hyperplasia in the lamina propria. Modulation in the expression levels of interferon γ, interleukin IL-10 and the Toll-like receptor 2 receptor. Decrease in the percentage of injury incidence, intestinal injury score and injury severity.	[104]
	*L. acidophilus* CGMCC 1.1878 and *L. fermentum* CGMCC 1.2029	In in vitro assays, both strains degraded *C*. *perfringens* α-toxin at 2 and 4 h of incubation. The pretreatment of *C*. *perfringens* with *L. acidophilus* significantly decreased (P ˂ 0.05) the percentage of adhesion of the pathogen to chicken intestinal epithelial cells. The relative expression levels of interleukins 6, 8 and 1β, inducible nitric oxide synthase and tumor necrosis factor α (TNF-α) were under-expressed in cells treated with *Lactobacillus* strains.	[105]
	*L. plantarum* R1.0320	Increase in the villus height: crypt depth ratio. Greater expression of MUC2 and a decrease in the expression of TNF-α in the mucosa of the ileum. Significant increase in the levels of IgA and IgG (or IgY) (on the 3rd day of administration) and IgM (on the 10th day of administration).	[106]
***Enterococcus***	*E. faecium* NCIMB 11181	Significant improvement in weight gain. Lower rate of intestinal lesions, histopathological inflammation, and apoptosis in intestinal cells. Overexpression of the gene encoding Claudin-1 that promotes epithelial cell attachment. Promote a balance in the intestinal immune response by modulating the expression of pro and anti-inflammatory cytokines, growth factors, heat shock proteins, and negative regulators of signaling mediated by Toll-like receptors. Modulation of the intestinal microbiota.	[107]

**Table 6:** Probiotic strains used for microbiota modulation to control necrotic enteritis caused by *Clostridium perfringens* in broiler chickens.

Probiotic	Concentration on the Diet	Time	Results	Reference
***Bacillus subtilis*** **CGMCC 1.921**	1 × 10^7^ and 1 × 10^8^ CFU/g	1 to 24 weeks	Significantly lower enumeration of *C. perfringens* in ceca digesta (*p* ˂ 0.05).	[108]
***Bacillus subtilis*** **PB6**	5 × 10^11^ CFU/kg	35 days	Reduced the intestinal *C. perfringens* enumeration significantly (*p* ˂ 0.05) and improved villi length by 10.88 and 30.46% (*p* ˂ 0.05) compared with uninfected and infected control groups.	[109]
***L. acidophilus*** **D2/CSL CECT 4529 and *B. subtilis* PB6 ATCC-PTA 6737**	0.1% of *L. acidophilus* (Lactomalt D2 Bio^®^); 0.05% of *B. subtilis* (Clostat^®^ brand dry—740210)	5 and 7 months	Significantly decrease in *E. coli*, *Clostridia*, and *Staphylococci* in cecum and ileum digesta (*p* ˂ 0.001). The two probiotic-supplemented diets, increased *Lactobacillus spp.* and *Bifidobacterium spp.* enumeration compared with the control diet.	[110]
**A mix of *Bacillus subtilis* DSM17299, *Clostridium butyricum*, and *Lactobacillus acidophilus***	2 × 10^2^ CFU/g and 4 × 10^2^ CFU/g	35 days	Significantly lower enumeration of *C. perfringens* and *Escherichia coli* in caecum and increase the enumeration of *Lactobacillus* and *Bifidobacterium*.	[111]
***Enterococcus faecium*** **NCIMB 11181**	1 × 10^6^ CFU/kg	26 days	Microbial community composition among the different groups, indicating significant variability in their microbial profiles. Highest relative abundance of *Lactobacillus* and *Butyricicoccus* in the cecum compared to the negative control and the *C. perfringens*-infected group without the administration of probiotic.	[107]
***L. johnsonii***	Feed and water delivery > 10^6^ CFU/g or mL; oral and litter delivery > 10^8^ CFU/mL of PBS	21 days	Establishment of the probiotic *L. johnsonii* in the intestinal tract. No statistically significant differences between delivery methods on the gut microbiota. Significantly decrease enterobacteria and *C. perfringens* in the ileum.	[112]

**Table 7:** Effect of the use of symbiotics on health, production parameters and the elimination of *Clostridium perfringens* in broilers.

Synbiotic Composition	Dose	Time	Results	Reference
***Enterococcus faecium*** **+ FOS + phycophytic substances**	1 kg/ton of feed	3 weeks	Decrease in mortality rate. Significant improvement (*p* ˂ 0.05) in the intestinal lesion score. Absence and reduction of histopathological alterations. Significant decrease (*p* ˂ 0.05) in the counts of *C*. *perfringens* in intestine and cecum, from day 3 to day 21, all this between the control group and the infected group fed with the synbiotic.	[138]
***Saccharomyces cerevisiae, Enterococcus faecium*** **, and** ***Bacillus*** **spp. (Avi-Lution^®^)**	1 and 2 g/Kg of feed	42 days	Significant increase in weight gain. Decrease in the percentage of mortality and in cumulative mortality at day 28 and 42 (both levels of synbiotic supplementation). No effect on intestinal lesions was observed.	[139]
**Synbiotic mix Kurago Biotek, 1 mL contains (7 log UFC/g of *Lactobacillus rhamnosus* HN001, *Pediococcus acidilactici* MA18/5M and 4.5% *Agave tequilana* fructans)**	50 µL/day	39 and 42 days	Increase in lactic acid bacteria enumerations in the duodenum. Improvement in intestinal morphology (higher villi and shallow crypts) in the duodenal mucosa.	[75]
***L. reuteri, E. faecium, B. animalis,*** **and *P. acidilactici* con FOS.**	0.05%	21 and 42 days	Significant difference in the height of the jejunal villi (*p* ˂ 0.05) on day 28 and 42. Significant weight gain (*p* ˂ 0.01) (at 21 and 42 days of the experiment). Significant decrease in *C*. *perfringens* enumerations from day 28 to day 42. Increasing the number of specific antibodies (IgA) at the level of the ceca mucosa.	[140]

**Table 8:** Phytogenics used in the control of Clostridium perfringens in broilers.

Product	Species	Results	Reference
Anise essential oil	*Pimpinella anisum*	Promotes intestinal development (longer villi and shallow crypts). Decreased intensity of intestinal lesions associated with necrotic enteritis.	[144]
Benzophenanthridine (alkaloids)	*Chelidonium majus*	Improves productive efficiency parameters. Reduces intestinal lesions and mortality associated with necrotic enteritis.	[145]
Oregano essential oil	*Origanum vulgare*	Increase in the body weight and breast weight at 42 d and promotes the cell proliferation in duodenum (P = 0.001) and jejunum (P = 0.012). Significantly decrease in the *Clostridium* counts. Decrease of gut lesions caused by *C. perfringens* and improved villus height to crypt depth, improvement of feed conversion efficiency. Increase of serum antibody titers and tendency to elevate occludin mRNA expression at the same time that linearly inhibited the mRNA expression of TLR-2 and tumor necrotic factor-α in the ileum.	[146,147,148]
Carvacrol	*Origanum vulgare*	Improved health (longer villi and shallow crypts) and function of the intestinal barrier. They promote intestinal colonization by *Bifidobacterium*. Antimicrobial activity against *C. perfringens* and reduction of intestinal lesions associated with necrotic enteritis.	[141,142,149,150]
Curcumin	*Curcuma Longa*	Decreases *C. perfringens* enumerations in intestinal contents.	[143]
Piperine	*Piper nigrum*	Decreases *C. perfringens* enumerations in intestinal contents.	[143]
Protopine (alkaloids)	*Eschscholzia californica Fumaria officinalis*	Improves productive efficiency parameters. Reduces intestinal lesions and mortality associated with necrotic enteritis.	[145]
Tannins	*Castanea sativa*	Inhibits the growth of *C. perfringens* in vitro and in vivo, without affecting food consumption and weight gain.	[151]
Thymol	*Thymus vulgaris*	Improved health (longer villi and shallow crypts) and function of the intestinal barrier. Promotes intestinal colonization by Bifidobacterium. Antimicrobial activity against *C. perfringens*.	[142,149,150]
Sanguinarin	*Chelidonium majus*	Improves productive efficiency parameters. Reduces intestinal lesions and mortality associated with necrotic enteritis.	[145]

## References

[1] Sarker M.R., Shivers R.P., Sparks S.G., Juneja V.K., McClane B.A. (2000). Comparative experiments to examine the effects of heating on vegetative cells and spores of *Clostridium perfringens* isolates carrying plasmid genes versus chromosomal enterotoxin genes. Appl. Environ. Microbiol..

[2] Juneja V.K., Novak J.S., Labbe R.J., Juneja V.K., Sofos J.N. (2010). Clostridium perfringens. Pathogens and Toxins in Foods: Challenges and Interventions.

[3] Mcclane B.A., Uzal F.A., Fernandez M., Lyerly D., Wilkins T., Dworkin M., Falkow S., Rosenberg E., Schleifer K.-H., Stackebrandt E. (2006). The Enterotoxic Clostridia. The Prokaryotes. Bacteria: Firmicutes, Cyanobacteria.

[4] Al-Khaldi S., Lampel K.A., Al-Khaldi S., Cahill S.M. (2012). Clostridium perfringens, phytohaemagglutinin (kidney bean lectin),Yersinia species. Bad Bug Book. Foodborne Pathogenic Microorganisms and Natural Toxins.

[5] Geissmann T.A., Teuber M., Meile L. (1999). Transcriptional analysis of the rubrerythrin and superoxide dismutase genes of *Clostridium perfringens*. J. Bacteriol..

[6] Jean D., Briolat V., Reysset G. (2004). Oxidative stress response in *Clostridium perfringens*. Microbiology.

[7] Johansson A., Aspan A., Bagge E., Båverud V., Engström B.E., Johansson K.E. (2006). Genetic diversity of *Clostridium perfringens* type A isolates from animals, food poisoning outbreaks and sludge. BMC Microbiol..

[8] Saito M. (1990). Production of enterotoxin by *Clostridium perfringens* derived from humans, animals, foods, and the natural environment in Japan. J. Food Prot..

[9] Grass J., Gould L.H., Mahon B. (2013). Epidemiology of Foodborne Disease Outbreaks Caused by *Clostridium perfringens*, United States, 1998–2010. Foodborne Pathog. Dis..

[10] Mellou K., Kyritsi M., Chrysostomou A., Sideroglou T., Georgakopoulou T., Hadjichristodoulou C. (2019). *Clostridium perfringens* foodborne outbreak during an athletic event in northern Greece, June 2019. Int. J. Environ. Res. Public Health.

[11] Monma C., Hatakeyama K., Obata H., Yokoyama K., Konishi N., Itoh T., Kai A. (2015). Four foodborne disease outbreaks caused by a new type of enterotoxin-producing *Clostridium perfringens*. J. Clin. Microbiol..

[12] Shaltout A., Zakaria M., Nabil E. (2017). Detection and typing of *Clostridium perfringens* in some retail chicken meat products. Benha Vet. Med. J..

[13] Lee C.A., Labbé R. (2018). Distribution of enterotoxin- and epsilon-positive *Clostridium perfringens* spores in U.S. retail spices. J. Food Prot..

[14] Butler A.J., Thomas M.K., Pintar K.D.M. (2015). Expert elicitation as a means to attribute 28 enteric pathogens to foodborne, waterborne, animal contact, and person-to-person transmission routes in Canada. Foodborne Pathog. Dis..

[15] Fohler S., Klein G., Hoedemaker M., Scheu T., Seyboldt C., Campe A., Jensen K.C., Abdulmawjood A. (2016). Diversity of *Clostridium perfringens* toxin-genotypes from dairy farms. BMC Microbiol..

[16] (2019). Centers for Disease Control and Prevention (CDC) Surveillance for Foodborne Disease Outbreaks United States, 2017: Annual Report.

[17] May F.J., Polkinghorne B.G., Fearnley E.J. (2016). Epidemiology of bacterial toxin—mediated foodborne gastroenteritis outbreaks in Australia, 2001 to 2013. CDI.

[18] Makkar H.P.S., Ankers P. (2014). Towards sustainable animal diets: A survey-based study. Anim. Feed Sci. Technol..

[19] Kay S., Edwards J., Brown J., Dixon R. (2019). Galleria mellonella infection model identifies both high and low lethality of *Clostridium perfringens* toxigenic strains and their response to antimicrobials. Front. Microbiol..

[20] Kiu R., Hall L.J. (2018). An update on the human and animal enteric pathogen *Clostridium perfringens*. Emerg. Microbes Infect..

[21] Rood J.I., Adams V., Lacey J., Lyras D., Mcclane B.A., Stephen B., Moore R.J., Popoff M.R., Sarker M.R., Songer J.G. (2018). Expansion of the *Clostridium perfringens* toxin-based typing scheme. Anaerobe.

[22] Uzal F.A., Freedman J.C., Shrestha A., Theoret J.R., Garcia J., Awad M.M., Adams V., Moore R.J., Rood J.I., Mcclane B.A. (2014). Towards an understanding of the role of *Clostridium perfringens* toxins in human and animal disease. Future Microbiol..

[23] Uzal F.A., Navarro M.A., Li J.J., Freedman J.C., Shrestha A., McClane B.A. (2018). Comparative pathogenesis of enteric clostridial infections in humans and animals. Anaerobe.

[24] Sayeed S., Uzal F.A., Fisher D.J., Saputo J., Vidal J.E., Chen Y., Gupta P., Rood J.I., McClane B.A. (2008). Beta toxin is essential for the intestinal virulence of *Clostridium perfringens* type C disease isolate CN3685 in a rabbit ileal loop model. Mol. Microbiol..

[25] Ghoneim N.H., Hamza D.A. (2017). Epidemiological studies on *Clostridium perfringens* food poisoning in retail foods. Rev. Sci. Tech. Off. Int. Epiz.

[26] Shrestha A., Uzal F.A., McClane B.A. (2018). Enterotoxic Clostridia: *Clostridium perfringens* Enteric Diseases. Gram-Positive Pathog..

[27] Miyamoto K., Li J., McClane B.A. (2012). Enterotoxigenic *Clostridium perfringens*: Detection and identification. Microbes Environ..

[28] Villarruel-López A., Ruíz-Quezada S.L., Castro-Rosas J., Gomez-Aldapa C.A., Olea-Rodríguez M.A., Nuño K., Navarro-Hidalgo V., Torres-Vitela M.R. (2016). Behavior and inactivation of enterotoxin-positive *Clostridium perfringens* in pork picadillo and tamales filled with pork picadillo under different cooking, storage, and reheating conditions. J. Food Prot..

[29] DuPont H.L. (2009). Bacterial Diarrhea. N. Engl. J. Med..

[30] Wong S., Santullo P., O’driscoll J., Jamous A., Hirani S.P., Saif M. (2017). Use of antibiotic and prevalence of antibiotic-associated diarrhoea in-patients with spinal cord injuries: A UK national spinal injury centre experience. Spinal Cord.

[31] Gui L., Subramony C., Fratkin J., Hughson M.D. (2002). Fatal enteritis necroticans (Pigbel) in a diabetic adult. Mod. Pathol..

[32] Stevens D.L., Bryant A.E. (2017). Necrotizing soft-tissue infections. N. Engl. J. Med..

[33] Zúñiga-Pereira A.M., Santamaría C., Gutierrez J.M., Alape-Girón A., Flores-Díaz M. (2019). Deficient skeletal muscle regeneration after injury induced by a *Clostridium perfringens* strain associated with gas gangrene. Infect. Immun..

[34] Zahoor I., Ghayas A., Basheer A. (2018). Genetics and genomics of susceptibility and immune response to necrotic enteritis in chicken: A review. Mol. Biol. Rep..

[35] Annett C.B., Viste J.R., Chirino-Trejo M., Classen H.L., Middleton D.M., Simko E. (2002). Necrotic enteritis: Effect of barley, wheat and corn diets on proliferation of *Clostridium perfringens* type A. Avian Pathol..

[36] Mejia D.B., Peñuela -S.L.M., Sanmiguel R.A. (2018). El gran impacto de *Clostridium perfringens* en aves de corral. Pubvet.

[37] Van Immerseel F., De Buck J., Pasmans F., Huyghebaert G., Haesebrouck F., Ducatelle R. (2004). *Clostridium perfringens* in poultry: An emerging threat for animal and public health. Avian Pathol..

[38] Gharib-Naseri K., Kheravii S.K., Keerqin C., Morgan N., Swick R.A., Choct M., Wu S.B. (2019). Two different *Clostridium perfringens* strains produce different levels of necrotic enteritis in broiler chickens. Poult. Sci..

[39] Timbermont L., Haesebrouck F., Ducatelle R., Van Immerseel F. (2011). Necrotic enteritis in broilers: An updated review on the pathogenesis. Avian Pathol..

[40] Timbermont L., Lanckriet A., Dewulf J., Nollet N., Schwarzer K., Haesebrouck F., Ducatelle R., van Immerseel F. (2010). Control of clostridium perfringens-induced necrotic enteritis in broilers by target-released butyric acid, fatty acids and essential oils. Avian Pathol..

[41] Rood J.I., Keyburn A.L., Moore R.J. (2016). NetB and necrotic enteritis: The hole movable story. Avian Pathol..

[42] Van Immerseel F., Rood J.I., Moore R.J., Titball R.W. (2009). Rethinking our understanding of the pathogenesis of necrotic enteritis in chickens. Trends Microbiol..

[43] Moore R.J. (2016). Necrotic enteritis predisposing factors in broiler chickens. Avian Pathol..

[44] Timbermont L., De Smet L., Van Nieuwerburgh F., Parreira V.R., Van Driessche G., Haesebrouck F., Ducatelle R., Prescott J., Deforce D., Devreese B. (2014). Perfrin, a novel bacteriocin associated with netB positive Clostridium perfringens strains from broilers with necrotic enteritis. Vet. Res..

[45] Flores-Díaz M., Barquero-Calvo E., Ramírez M., Alape-Girón A. (2016). Role of *Clostridium perfringens* Toxins in Necrotic Enteritis in Poultry. Microb. Toxins.

[46] Prescott J.F., Parreira V.R., Mehdizadeh Gohari I., Lepp D., Gong J. (2016). The pathogenesis of necrotic enteritis in chickens: What we know and what we need to know: A review. Avian Pathol..

[47] Razmyar J., Peighambari S.M., Zamani A.H. (2017). Detection of a Newly Described Bacteriocin, Perfrin, among *Clostridium perfringens* Isolates from Healthy and Diseased Ostriches and Broiler Chickens in Iran. Avian Dis..

[48] Wade B., Keyburn A.L., Seemann T., Rood J.I., Moore R.J. (2015). Binding of *Clostridium perfringens* to collagen correlates with the ability to cause necrotic enteritis in chickens. Vet. Microbiol..

[49] Cheung J.K., Keyburn A.L., Carter G.P., Lanckriet A.L., Van Immerseel F., Moore R.J., Rood J.I. (2010). The VirSR two-component signal transduction system regulates NetB toxin production in *Clostridium perfringens*. Infect. Immun..

[50] Yu Q., Lepp D., Gohari I.M., Wu T., Zhou H., Yin X., Yu H., Prescott J.F., Nie S.P., Xie M.Y. (2017). The Agr-like quorum sensing system is required for pathogenesis of necrotic enteritis caused by *Clostridium perfringens* in poultry. Infect. Immun..

[51] Wang Y.H. (2020). Sialidases From *Clostridium perfringens* and Their Inhibitors. Front. Cell. Infect. Microbiol..

[52] Lepp D., Roxas B., Parreira V.R., Marri P.R., Rosey E.L., Gong J., Songer J.G., Vedantam G., Prescott J.F. (2010). Identification of novel pathogenicity loci in *Clostridium perfringens* strains that cause Avian necrotic enteritis. PLoS ONE.

[53] Savva C.G., Da Costa S.P.F., Bokori-Brown M., Naylor C.E., Cole A.R., Moss D.S., Titball R.W., Basak A.K. (2013). Molecular architecture and functional analysis of NetB, a pore-forming toxin from *Clostridium perfringens*. J. Biol. Chem..

[54] Popoff M.R., Bouvet P. (2009). Clostridial toxins. Future Microbiol..

[55] Navarro M.A., McClane B.A., Uzal F.A. (2018). Mechanisms of action and cell death associated with *Clostridium perfringens* toxins. Toxins.

[56] Paiva D., McElroy A. (2014). Necrotic enteritis: Applications for the poultry industry. J. Appl. Poult. Res..

[57] Edelblum K.L., Turner J.R. (2009). The Tight Junction in Inflammatory Disease: Communication Breakdown. Curr. Opin. Pharmacol..

[58] Llanco L.A., Nakano V., Moraes C.T.P.d., Piazza R.M.F., Avila-Campos M.J. (2017). Adhesion and invasion of *Clostridium perfringens* type A into epithelial cells. Brazilian J. Microbiol..

[59] Coursodon C.F., Glock R.D., Moore K.L., Cooper K.K., Songer J.G. (2012). TpeL-producing strains of *Clostridium perfringens* type A are highly virulent for broiler chicks. Anaerobe.

[60] Schorch B., Song S., Van Diemen F.R., Bock H.H., May P., Herz J., Brummelkamp T.R., Papatheodorou P., Aktories K. (2014). LRP1 is a receptor for *Clostridium perfringens* TpeL toxin indicating a two-receptor model of clostridial glycosylating toxins. Proc. Natl. Acad. Sci. USA.

[61] Alshamy Z., Richardson K.C., Hünigen H., Hafez H.M., Plendl J., Al Masri S. (2018). Comparison of the gastrointestinal tract of a dual-purpose to a broiler chicken line: A qualitative and quantitative macroscopic and microscopic study. PLoS ONE.

[62] Laledashti M.A., Saki A.A., Rafati A.A., Abdolmaleki M. (2020). Effect of in-ovo feeding of iron nanoparticles and methionine hydroxy analogue on broilers chickens small intestinal characteristics. Acta Sci. Anim. Sci..

[63] Sun X., Jia Z. (2018). Microbiome modulates intestinal homeostasis against inflammatory diseases. Vet. Immunol. Immunopathol..

[64] Cooper K.K., Songer J.G., Uzal F.A. (2013). Diagnosing clostridial enteric disease in poultry. J. Vet. Diagnostic Investig..

[65] Redondo L.M., Redondo E.A., Delgado F., La Sala L.F., Fernández Miyakawa M.E. (2016). An Experimental Reproduction of Necrotic Enteritis in Broiler Chickens. J. Vet. Sci. Med..

[66] Smyth J.A. (2016). Pathology and diagnosis of necrotic enteritis: Is it clear-cut?. Avian Pathol..

[67] Awad W.A., Hess C., Hess M. (2017). Enteric pathogens and their toxin-induced disruption of the intestinal barrier through alteration of tight junctions in chickens. Toxins.

[68] De Meyer F., Eeckhaut V., Ducatelle R., Dhaenens M., Daled S., Dedeurwaerder A., De Gussem M., Haesebrouck F., Deforce D., Van Immerseel F. (2019). Host intestinal biomarker identification in a gut leakage model in broilers. Vet. Res..

[69] Perez-Lopez A., Behnsen J., Nuccio S.P., Raffatellu M. (2016). Mucosal immunity to pathogenic intestinal bacteria. Nat. Rev. Immunol..

[70] Gholamiandehkordi A.R., Timbermont L., Lanckriet A., Van Den Broeck W., Pedersen K., Dewulf J., Pasmans F., Haesebrouck F., Ducatelle R., Van Immerseel F. (2007). Quantification of gut lesions in a subclinical necrotic enteritis model. Avian Pathol..

[71] Goossens E., Valgaeren B.R., Pardon B., Haesebrouck F., Ducatelle R., Deprez P.R., Van Immerseel F. (2017). Rethinking the role of alpha toxin in *Clostridium perfringens*-associated enteric diseases: A review on bovine necro-haemorrhagic enteritis. Vet. Res..

[72] Sanchez L.C. (2018). Disorders of the Gastrointestinal System. Equine Intern. Med..

[73] Clavijo V., Flórez M.J.V. (2018). The gastrointestinal microbiome and its association with the control of pathogens in broiler chicken production: A review. Poult. Sci..

[74] Lacey J.A., Stanley D., Keyburn A.L., Ford M., Chen H., Johanesen P., Lyras D., Moore R.J. (2018). *Clostridium perfringens*-mediated necrotic enteritis is not influenced by the pre-existing microbiota but is promoted by large changes in the post-challenge microbiota. Vet. Microbiol..

[75] Villagran-de la Mora Z., Nuño K., Olga V., Avalos H., Castro-rosas J., Carlos G., Angulo C., Ascencio F. (2019). Effect of a Synbiotic Mix on Intestinal Structural Changes, and Salmonella Typhimurium and *Clostridium Perfringens* Colonization in Broiler Chickens. Animals.

[76] Keyburn A.L., Sheedy S.A., Ford M.E., Williamson M.M., Awad M.M., Rood J.I., Moore R.J. (2006). Alpha-toxin of *Clostridium perfringens* is not an essential virulence factor in necrotic enteritis in chickens. Infect. Immun..

[77] Shojadoost B., Vince A.R., Prescott J.F. (2012). The successful experimental induction of necrotic enteritis in chickens by *Clostridium perfringens*: A critical review. Vet. Res..

[78] Chuang W.Y., Lin W.C., Hsieh Y.C., Huang C.M., Chang S.C., Lee T.T. (2019). Evaluation of the combined use of Saccharomyces cerevisiae and Aspergillus oryzae with phytase fermentation products on growth, inflammatory, and intestinal morphology in broilers. Animals.

[79] Lee K.W., Lillehoj H.S., Jeong W., Jeoung H.Y., An D.J. (2011). Avian necrotic enteritis: Experimental models, host immunity, pathogenesis, risk factors, and vaccine development. Poult. Sci..

[80] Lee Y., Kim W.H., Lee S.J., Lillehoj H.S. (2018). Detection of chicken interleukin-10 production in intestinal epithelial cells and necrotic enteritis induced by *Clostridium perfringens* using capture ELISA. Vet. Immunol. Immunopathol..

[81] Wise M.G., Siragusa G.R. (2005). Quantitative detection of *Clostridium perfringens* in the broiler fowl gastrointestinal tract by real-time PCR. Appl. Environ. Microbiol..

[82] Bailey M.A., Macklin K.S., Krehling J.T. (2013). Use of a Multiplex PCR for the Detection of Toxin-Encoding Genes netB and tpeL in Strains of *Clostridium perfringens*. ISRN Vet. Sci..

[83] Food and Agriculture Organization, (FAO), World Health Organization, (WHO), FAO, WHO (2006). Probiotics in Food Health and Nutritional Properties and Guidelines for Evaluation.

[84] Jin L.Z., Ho Y.W., Abdullah N., Jalaludin S. (1997). Probiotics in poultry: Modes of action. Worlds. Poult. Sci. J..

[85] Park Y.H., Hamidon F., Rajangan C., Soh K.P., Gan C.Y., Lim T.S., Abdullah W.N.W., Liong M.T. (2016). Application of probiotics for the production of safe and high-quality poultry meat. Korean J. Food Sci. Anim. Resour..

[86] Ma T., Suzuki Y., Guan L.L. (2018). Dissect the mode of action of probiotics in affecting host-microbial interactions and immunity in food producing animals. Vet. Immunol. Immunopathol..

[87] Azad M.A.K., Sarker M., Li T., Yin J. (2018). Probiotic Species in the Modulation of Gut Microbiota: An Overview. Biomed Res. Int..

[88] Popova T. (2017). Effect of probiotics in poultry for improving meat quality. Curr. Opin. Food Sci..

[89] Carlander D., Stålberg J., Larsson A. (1999). Chicken antibodies: A clinical chemistry perspective. Ups. J. Med. Sci..

[90] Khalique A., Zeng D., Shoaib M., Wang H., Qing X., Rajput D.S., Pan K., Ni X. (2020). Probiotics mitigating subclinical necrotic enteritis (SNE) as potential alternatives to antibiotics in poultry. AMB Express.

[91] Menconi A., Sokale A.O., Mendoza S.M., Whelan R., Doranalli K. (2020). Effect of Bacillus subtilis DSM 32315 under different Necrotic Enteritis models in broiler chickens: A meta-analysis of 5 independent research trials. Avian Dis..

[92] Johnson T.J., Youmans B.P., Noll S., Cardona C., Evans N.P., Peter Karnezos T., Ngunjiri J.M., Abundo M.C., Lee C.W. (2018). A consistent and predictable commercial broiler chicken bacterial microbiota in antibiotic-free production displays strong correlations with performance. Appl. Environ. Microbiol..

[93] Stanley D., Hughes R.J., Geier M.S., Moore R.J. (2016). Bacteria within the gastrointestinal tract microbiota correlated with improved growth and feed conversion: Challenges presented for the identification of performance enhancing probiotic bacteria. Front. Microbiol..

[94] La Ragione R.M., Narbad A., Gasson M.J., Woodward M.J. (2004). In vivo characterization of Lactobacillus johnsonii FI9785 for use as a defined competitive exclusion agent against bacterial pathogens in poultry. Lett. Appl. Microbiol..

[95] Gervasi T., Lo Curto R., Minniti E., Narbad A., Mayer M.J. (2014). Application of Lactobacillus johnsonii expressing phage endolysin for control of *Clostridium perfringens*. Lett. Appl. Microbiol..

[96] Kim C.H., Shin K.S., Woo K.C., Paik I.K. (2009). Effect of Dietary Oligosaccharides on the Performance, Intestinal Microflora and Serum Immunoglobulin Contents in Laying Hens. Korean J. Poult. Sci..

[97] Wu Y., Shao Y., Song B., Zhen W., Wang Z., Guo Y., Shahid M.S., Nie W. (2018). Effects of Bacillus coagulans supplementation on the growth performance and gut health of broiler chickens with *Clostridium perfringens*-induced necrotic enteritis. J. Anim. Sci. Biotechnol..

[98] Xu S., Lin Y., Zeng D., Zhou M., Zeng Y., Wang H., Zhou Y., Zhu H., Pan K., Jing B. (2018). Bacillus licheniformis normalize the ileum microbiota of chickens infected with necrotic enteritis. Sci. Rep..

[99] Zhao Y., Zeng D., Wang H., Qing X., Sun N., Xin J., Luo M., Khalique A., Pan K., Shu G. (2019). Dietary Probiotic Bacillus licheniformis H2 Enhanced Growth Performance, Morphology of Small Intestine and Liver, and Antioxidant Capacity of Broiler Chickens Against *Clostridium perfringens*–Induced Subclinical Necrotic Enteritis. Probiotics Antimicrob. Proteins.

[100] Aljumaah M.R., Alkhulaifi M.M., Abudabos A.M., Aljumaah R.S., Alsaleh A.N., Stanley D. (2020). Bacillus subtilis PB6 based probiotic supplementation plays a role in the recovery after the necrotic enteritis challenge. PLoS ONE.

[101] Ramlucken U., Ramchuran S.O., Moonsamy G., Lalloo R., Thantsha M.S., Jansen van Rensburg C. (2020). A novel Bacillus based multi-strain probiotic improves growth performance and intestinal properties of *Clostridium perfringens* challenged broilers. Poult. Sci..

[102] Wang H., Ni X., Qing X., Liu L., Xin J., Luo M., Khalique A., Dan Y., Pan K., Jing B. (2018). Probiotic lactobacillus johnsonii BS15 improves blood parameters related to immunity in broilers experimentally infected with subclinical necrotic enteritis. Front. Microbiol..

[103] Cao L., Wu X.H., Bai Y.L., Wu X.Y., Gu S.B. (2019). Anti-inflammatory and antioxidant activities of probiotic powder containing Lactobacillus plantarum 1.2567 in necrotic enteritis model of broiler chickens. Livest. Sci..

[104] Cao L., Yang X.J., Li Z.J., Sun F.F., Wu X.H., Yao J.H. (2012). Reduced lesions in chickens with *Clostridium perfringens*-induced necrotic enteritis by Lactobacillus fermentum 1.2029. Poult. Sci..

[105] Guo S., Liu D., Zhang B., Li Z., Li Y., Ding B., Guo Y. (2017). Two Lactobacillus species inhibit the growth and α-toxin production of *Clostridium perfringens* and induced proinflammatory factors in chicken intestinal epithelial cells in vitro. Front. Microbiol..

[106] Xu T., Chen Y., Yu L., Wang J., Huang M., Zhu N. (2020). Effects of Lactobacillus plantarum on intestinal integrity and immune responses of egg-laying chickens infected with *Clostridium perfringens* under the free-range or the specific pathogen free environment. BMC Vet. Res..

[107] Wu Y., Zhen W., Geng Y., Wang Z., Guo Y. (2019). Pretreatment with probiotic Enterococcus faecium NCIMB 11181 ameliorates necrotic enteritis-induced intestinal barrier injury in broiler chickens. Sci. Rep..

[108] Guo J.R., Dong X.F., Liu S., Tong J.M. (2017). Effects of long-term Bacillus subtilis CGMCC 1.921 supplementation on performance, egg quality, and fecal and cecal microbiota of laying hens. Poult. Sci..

[109] Jayaraman S., Thangavel G., Kurian H., Mani R., Mukkalil R., Chirakkal H. (2013). Bacillus subtilis PB6 improves intestinal health of broiler chickens challenged with *Clostridium perfringens*-induced necrotic enteritis. Poult. Sci..

[110] Forte C., Acuti G., Manuali E., Casagrande Proietti P., Pavone S., Trabalza-Marinucci M., Moscati L., Onofri A., Lorenzetti C., Franciosini M.P. (2016). Effects of two different probiotics on microflora, morphology, and morphometry of gut in organic laying hens. Poult. Sci..

[111] Hossain M.M., Begum M., Kim I.H. (2015). Effect of Bacillus subtilis, Clostridium butyricum and Lactobacillus acidophilus endospores on growth performance, nutrient digestibility, meat quality, relative organ weight, microbial shedding and excreta noxious gas emission in broilers. Vet. Med..

[112] Olnood C.G., Beski S.S.M., Iji P.A., Choct M. (2015). Delivery routes for probiotics: Effects on broiler performance, intestinal morphology and gut microflora. Anim. Nutr..

[113] Rantala M., Nurmi E. (1973). Prevention of the growth of salmonella infantis in chicks by the flora of the alimentary tract of chickens. Br. Poult. Sci..

[114] Caly D.L., D’Inca R., Auclair E., Drider D. (2015). Alternatives to antibiotics to prevent necrotic enteritis in broiler chickens: A microbiologist’s perspective. Front. Microbiol..

[115] Craven A.S.E., Stern N.J., Cox N.A., Bailey J.S., Berrang M., Craven S.E., Stern N.J., Cox N.A., Bailey J.S., Berrang M. (2018). Cecal Carriage of Clostridium perfringens in Broiler Chickens Given Mucosal Starter Culture. Avian Dis..

[116] Rubio L.A. (2019). Possibilities of early life programming in broiler chickens via intestinal microbiota modulation. Poult. Sci..

[117] Ghareeb K., Awad W.A., Mohnl M., Porta R., Biarnés M., Böhm J., Schatzmayr G. (2012). Evaluating the efficacy of an avian-specific probiotic to reduce the colonization of Campylobacter jejuni in broiler chickens. Poult. Sci..

[118] Froebel L.K., Jalukar S., Lavergne T.A., Lee J.T., Duong T. (2019). Administration of dietary prebiotics improves growth performance and reduces pathogen colonization in broiler chickens. Poult. Sci..

[119] Kim S.A., Jang M.J., Kim S.Y., Yang Y., Pavlidis H.O., Ricke S.C. (2019). Potential for prebiotics as feed additives to limit foodborne Campylobacter establishment in the poultry gastrointestinal tract. Front. Microbiol..

[120] Bucław M. (2017). Inulin in poultry production. Worlds. Poult. Sci. J..

[121] Teng P., Kim W.K. (2018). Review: Roles of Prebiotics in Intestinal Ecosystem of Broilers. Front. Vet. Sci..

[122] Ghasemi H.A., Shivazad M., Mirzapour Rezaei S.S., Torshizi M.A.K. (2015). Effect of synbiotic supplementation and dietary fat sources on broiler performance, serum lipids, muscle fatty acid profile and meat quality. Br. Poult. Sci..

[123] Huyghebaert G., Ducatelle R., Van Immerseel F. (2011). Van An update on alternatives to antimicrobial growth promoters for broilers. Vet. J..

[124] Hashim M.M., Arsenault R.J., Byrd J.A., Kogut M.H., Al-Ajeeli M., Bailey C.A. (2018). Influence of different yeast cell wall preparations and their components on performance and immune and metabolic pathways in *Clostridium perfringens*-challenged broiler chicks. Poult. Sci..

[125] Guaragni A., Boiago M.M., Bottari N.B., Morsch V.M., Lopes T.F., Schafer da Silva A. (2020). Feed supplementation with inulin on broiler performance and meat quality challenged with *Clostridium perfringens:* Infection and prebiotic impacts. Microb. Pathog..

[126] Ricke S.C., Lee S.I., Kim S.A., Park S.H., Shi Z. (2020). Prebiotics and the poultry gastrointestinal tract microbiome. Poult. Sci..

[127] Ajuwon K.M. (2016). Toward a better understanding of mechanisms of probiotics and prebiotics action in poultry species. J. Appl. Poult. Res..

[128] Kheravii S.K., Swick R.A., Choct M., Wu S.B. (2018). Effect of oat hulls as a free choice feeding on broiler performance, short chain fatty acids and microflora under a mild necrotic enteritis challenge. Anim. Nutr..

[129] Mtei A.W., Abdollahi M.R., Schreurs N., Girish C.K., Ravindran V. (2019). Dietary inclusion of fibrous ingredients and bird type influence apparent ileal digestibility of nutrients and energy utilization. Poult. Sci..

[130] Gibson G.R., Roberfroid M.B. (1995). Dietary modulation of the human colonic microbiota: Introducing the concept of prebiotics. J. Nutr..

[131] Peña A.S. (2007). Flora intestinal, probióticos, prebióticos, simbióticos y alimentos novedosos. Rev. Esp. Enferm. Dig..

[132] Pandey K.R., Naik S.R., Vakil B.V. (2015). Probiotics, prebiotics and synbiotics—A review. J. Food Sci. Technol..

[133] Gadde U., Kim W.H., Oh S.T., Lillehoj H.S. (2017). Alternatives to antibiotics for maximizing growth performance and feed efficiency in poultry: A review. Anim. Heal. Res. Rev..

[134] Markowiak P., Ślizewska K. (2018). The role of probiotics, prebiotics and synbiotics in animal nutrition. Gut Pathog..

[135] Baffoni L., Gaggìa F., Garofolo G., Di Serafino G., Buglione E., Di Giannatale E., Di Gioia D. (2017). Evidence of Campylobacter jejuni reduction in broilers with early synbiotic administration. Int. J. Food Microbiol..

[136] Koc F., Samli H., Okur A., Ozduven M., Akyurek H., Senkoylu N. (2010). Effects of Saccharomyces cerevisiae and/or mannanoligosaccharide on performance, blood parameters and intestinal microbiota of broiler chicks. Bulg. J. Agric. Sci..

[137] Mookiah S., Sieo C.C., Ramasamy K., Abdullah N., Ho Y.W. (2014). Effects of dietary prebiotics, probiotic and synbiotics on performance, caecal bacterial populations and caecal fermentation concentrations of broiler chickens. J. Sci. Food Agric..

[138] Abd El-Ghany W.A. (2010). Comparative evaluation on the effect of coccidiostate and synbiotic preparations on prevention of *Clostridium perfringens* in broiler chickens. Glob. Vet..

[139] Krueger L.A., Spangler D.A., Vandermyde D.R., Sims M.D., Ayangbile G.A. (2017). Avi-Lution^®^ supplemented at 1.0 or 2.0 g/kg in feed improves the growth performance of broiler chickens during challenge with bacitracin-resistant *Clostridium perfringens*. Poult. Sci..

[140] Shanmugasundaram R., Markazi A., Mortada M., Ng T.T., Applegate T.J., Bielke L.R., Syed B., Pender C.M., Curry S., Murugesan G.R. (2020). Research Note: Effect of synbiotic supplementation on caecal *Clostridium perfringens* load in broiler chickens with different necrotic enteritis challenge models. Poult. Sci..

[141] Yang C., Chowdhury M.A.K., Hou Y., Gong J. (2015). Phytogenic compounds as alternatives to in-feed antibiotics: Potentials and challenges in application. Pathogens.

[142] Pham V.H., Kan L., Huang J., Geng Y., Zhen W., Guo Y., Abbas W., Wang Z. (2020). Dietary encapsulated essential oils and organic acids mixture improves gut health in broiler chickens challenged with necrotic enteritis. J. Anim. Sci. Biotechnol..

[143] Granstad S., Kristoffersen A.B., Benestad S.L., Sjurseth S.K., David B., Sørensen L., Fjermedal A., Edvardsen D.H., Sanson G., Løvland A. (2020). Effect of feed additives as alternatives to in-feed antimicrobials on production performance and intestinal *Clostridium perfringens* counts in broiler chickens. Animals.

[144] Abudabos A.M. (2013). Use of a competitive exclusion product (Aviguard) to prevent *Clostridium perfringens* colonization in broiler chicken under induced challenge. Pak. J. Zool..

[145] Hussein E.O.S., Ahmed S.H., Abudabos A.M., Aljumaah M.R., Alkhlulaifi M.M., Nassan M.A., Suliman G.M., Naiel M.A.E., Swelum A.A. (2020). Effect of antibiotic, phytobiotic and probiotic supplementation on growth, blood indices and intestine health in broiler chicks challenged with *Clostridium perfringens*. Animals.

[146] Tzora A., Giannenas I., Karamoutsios A., Papaioannou N., Papanastasiou D., Bonos E., Skoufos S., Bartzanas T., Skoufos I. (2017). Effects of oregano, attapulgite, benzoic acid and their blend on chicken performance, intestinal microbiology and intestinal morphology. J. Poult. Sci..

[147] Kirkpinar F., Ünlü H.B., Özdemir G. (2011). Effects of oregano and garlic essential oils on performance, carcase, organ and blood characteristics and intestinal microflora of broilers. Livest. Sci..

[148] Du E., Wang W., Gan L., Li Z., Guo S., Guo Y. (2016). Effects of thymol and carvacrol supplementation on intestinal integrity and immune responses of broiler chickens challenged with Clostridium perfringens. J. Anim. Sci. Biotechnol..

[149] Abdelli N., Pérez J.F., Vilarrasa E., Luna I.C., Melo-Duran D., D’angelo M., Solà-Oriol D. (2020). Targeted-release organic acids and essential oils improve performance and digestive function in broilers under a necrotic enteritis challenge. Animals.

[150] Sun Q., Liu D., Guo S., Chen Y., Guo Y. (2015). Effects of dietary essential oil and enzyme supplementation on growth performance and gut health of broilers challenged by *Clostridium perfringens*. Anim. Feed Sci. Technol..

[151] Mannelli F., Minieri S., Tosi G., Secci G., Daghio M., Massi P., Fiorentini L., Galigani I., Lancini S., Rapaccini S. (2019). Effect of chestnut tannins and short chain fatty acids as anti-microbials and as feeding supplements in broilers rearing and meat quality. Animals.

[152] Dahiya J.P., Wilkie D.C., Van Kessel A.G., Drew M.D. (2006). Potential strategies for controlling necrotic enteritis in broiler chickens in post-antibiotic era. Anim. Feed Sci. Technol..

[153] Lensing M., van der Klis J.D., Fabri T., Cazemier A., Else A.J. (2010). Efficacy of a lactylate on production performance and intestinal health of broilers during a subclinical *Clostridium perfringens* infection. Poult. Sci..

[154] Drew M.D., Syed N.A., Goldade B.G., Laarveld B., Van Kessel A.G. (2004). Effects of dietary protein source and level on intestinal populations of *Clostridium perfringens* in broiler chickens. Poult. Sci..

[155] Kumar S., Adhikari P., Oakley B., Kim W.K. (2019). Changes in cecum microbial community in response to total sulfur amino acid (TSAA: DL-methionine) in antibiotic-free and supplemented poultry birds. Poult. Sci..

[156] Sieo C.C., Abdullah N., Tan W.S., Ho Y.W. (2005). Influence of β-glucanase-producing lactobacillus strains on intestinal characteristics and feed passage rate of broiler chickens. Poult. Sci..

[157] Tsiouris V., Georgopoulou I., Batzios C., Pappaioannou N., Ducatelle R., Fortomaris P. (2014). Temporary feed restriction partially protects broilers from necrotic enteritis. Avian Pathol..

[158] Wilkie D.C., Van Kessel A.G., White L.J., Laarveld B., Drew M.D. (2005). Dietary amino acids affect intestinal *Clostridium perfringens* populations in broiler chickens. Can. J. Anim. Sci..

[159] Singh Y., Amerah A.M., Ravindran V. (2014). Whole grain feeding: Methodologies and effects on performance, digestive tract development and nutrient utilisation of poultry. Anim. Feed Sci. Technol..

[160] Tsiouris V. (2016). Poultry management: A useful tool for the control of necrotic enteritis in poultry. Avian Pathol..

[161] Kiarie E., Romero L.F., Nyachoti C.M. (2013). The role of added feed enzymes in promoting gut health in swine and poultry. Nutr. Res. Rev..

[162] Barekatain M.R., Antipatis C., Rodgers N., Walkden-Brown S.W., Iji P.A., Choct M. (2013). Evaluation of high dietary inclusion of distillers dried grains with solubles and supplementation of protease and xylanase in the diets of broiler chickens under necrotic enteritis challenge. Poult. Sci..

[163] Wu Y.B., Ravindran V. (2004). Influence of whole wheat inclusion and xylanase supplementation on the performance, digestive tract measurements and carcass characteristics of broiler chickens. Anim. Feed Sci. Technol..

[164] Latorre J.D., Hernandez-Velasco X., Kuttappan V.A., Wolfenden R.E., Vicente J.L., Wolfenden A.D., Bielke L.R., Prado-Rebolledo O.F., Morales E., Hargis B.M. (2015). Selection of Bacillus spp. for cellulase and xylanase production as direct-fed microbials to reduce digesta viscosity and *Clostridium perfringens* proliferation using an in vitro digestive model in different poultry diets. Front. Vet. Sci..

[165] Choct M., Sinlae M., Al-Jassim R.A.M., Pettersson D. (2006). Effects of xylanase supplementation on between-bird variation in energy metabolism and the number of *Clostridium perfringens* in broilers fed a wheat-based diet. Aust. J. Agric. Res..

[166] Wang G., Wang Y., Yu L., Jiang Y., Liu J., Cheng Z. (2012). New pathogenetic characters of reticuloendotheliosis virus isolated from Chinese partridge in specific-pathogen-free chickens. Microb. Pathog..

[167] Borda-Molina D., Seifert J., Camarinha-Silva A. (2018). Current Perspectives of the Chicken Gastrointestinal Tract and Its Microbiome. Comput. Struct. Biotechnol. J..

